# *Candida auris* Isolates Resistant to Three Classes of Antifungal Medications — New York, 2019

**DOI:** 10.15585/mmwr.mm6901a2

**Published:** 2020-01-10

**Authors:** Belinda Ostrowsky, Jane Greenko, Eleanor Adams, Monica Quinn, Brittany O’Brien, Vishnu Chaturvedi, Elizabeth Berkow, Snigdha Vallabhaneni, Kaitlin Forsberg, Sudha Chaturvedi, Emily Lutterloh, Debra Blog, C. auris Investigation Work Group, C. auris Investigation Work Group, Coralie Bucher, Ronald Jean Denis, Richard Erazo, Rafael Fernandez, Karen Southwick, Yan Chun Zhu

**Affiliations:** ^1^Division of Healthcare Quality Promotion, National Center for Emerging and Zoonotic Infectious Diseases, CDC; ^2^Metropolitan Regional Office, New York State Department of Health, New York, New York; ^3^New York State Department of Health, Albany, New York; ^4^Wadsworth Laboratory, New York State Department of Health, Albany, New York; ^5^School of Public Health, State University of New York, Albany, New York; ^6^Division of Foodborne, Waterborne, and Environmental Diseases, National Center for Emerging and Zoonotic Infectious Diseases, CDC.; New York State Department of Health, Albany, New York;; Metropolitan Regional Office, New York State Department of Health, New York, New York; Metropolitan Regional Office, New York State Department of Health, New York, New York; New York State Department of Health, Metropolitan Regional Office, New York; Metropolitan Regional Office, New York State Department of Health, New York, New York; New York State Department of Health, Wadsworth Laboratory, Albany, New York.

*Candida auris* is a globally emerging yeast that causes outbreaks in health care settings and is often resistant to one or more classes of antifungal medications (*1*). Cases of *C. auris* with resistance to all three classes of commonly prescribed antifungal drugs (pan-resistance) have been reported in multiple countries (*1*). *C. auris* has been identified in the United States since 2016; the largest number (427 of 911 [47%]) of confirmed clinical cases reported as of October 31, 2019, have been reported in New York, where *C. auris* was first detected in July 2016 (*1*,*2*). As of June 28, 2019, a total of 801 patients with *C. auris* were identified in New York, based on clinical cultures or swabs of skin or nares obtained to detect asymptomatic colonization (*3*). Among these patients, three were found to have pan-resistant *C. auris* that developed after receipt of antifungal medications, including echinocandins, a class of drugs that targets the fungal cell wall. All three patients had multiple comorbidities and no known recent domestic or foreign travel. Although extensive investigations failed to document transmission of pan-resistant isolates from the three patients to other patients or the environment, the emergence of pan-resistance is concerning. The occurrence of these cases underscores the public health importance of surveillance for *C. auris*, the need for prudent antifungal prescribing, and the importance of conducting susceptibility testing on all clinical isolates, including serial isolates from individual patients, especially those treated with echinocandin medications. This report summarizes investigations related to the three New York patients with pan-resistant infections and the subsequent actions conducted by the New York State Department of Health and hospital and long-term care facility partners.

Clinical *C. auris* cases were defined as those in which *C. auris* was identified in a clinical culture obtained to diagnose or treat disease. Screening cases were defined as those in which *C. auris* was identified by polymerase chain reaction testing and culture, or by culture only, of a sample from an axilla, groin, or nares swab obtained for the purpose of state public health surveillance (*2*). To assess ongoing colonization with *C. auris,* additional swabs were collected over time from patients colonized with *C. auris*.

Wadsworth Center, the New York State public health laboratory, conducted testing to confirm presumptive *C. auris* isolates from various health care facilities in New York during August 2016–June 2019 by matrix-assisted laser desorption/ionization time-of-flight mass spectrometry, using both the manufacturer’s and in-house validated library databases. The laboratory also performed antifungal susceptibility testing for azoles and echinocandins by broth microdilution and for amphotericin B, by E-test methods* as described previously, and categorized isolates as resistant based on CDC’s tentative breakpoints (*1*,*2*). A pan-resistant isolate was defined as one with resistance to the triazole class (fluconazole minimum inhibitory concentration [MIC] ≥32 *μ*g/mL), polyene class (amphotericin B MIC ≥2 *μ*g/mL [E-test values of 1.5 rounded up to 2]), and echinocandins (anidulafungin MIC ≥4 *μ*g/mL, caspofungin MIC ≥2 *μ*g/mL, micafungin MIC ≥4 *μ*g/mL), tested at Wadsworth Center with confirmation by the laboratory at CDC’s Mycotic Diseases Branch (*1*,*2*).

Epidemiologic investigation of patients with pan-resistant cases included collecting clinical and exposure data, screening close contacts (persons who had an epidemiologic link to a patient in place or time), and assessing infection control practices in health care facilities that cared for the patients (*2*,*4*,*5*). When close contacts could be located, the New York State Department of Health attempted to obtain swabs for culture.

Site visits involved observations of infection control practices, on-site education, and point prevalence studies. During point prevalence surveys, samples were collected from the nares, axilla, and groin of consenting patients. When possible, samples from the environments of facilities where patients with pan-resistant infections were admitted or resided were collected, with priority given to frequently touched surfaces and objects in patients’ rooms.

As of June 28, 2019, a total of 801 patients with *C. auris* were detected in New York, identified through clinical cultures (349) or skin or nares screening swabs only (452) (*3*). Testing of the first available clinical isolates with susceptibilities revealed that 276 of 277 (99.6%) were resistant to fluconazole, 170 of 277 (61.3%) were resistant to amphotericin B, and none was resistant to echinocandins (*1*,*6*). Testing of subsequent available isolates obtained from infected patients with susceptibilities revealed 330 of 331 (99.7%) were resistant to fluconazole, 210 of 331 (63.4%) were resistant to amphotericin B, and 13 of 331 (3.9%) were resistant to echinocandins (*1*,*6*). Three patients’ subsequent isolates were pan-resistant.

The first two patients with pan-resistant *C. auris* infections (patient A and patient B) were aged >50 years and residents of long-term care facilities; each had multiple underlying medical conditions, including ventilator dependence and colonization with multidrug-resistant bacteria (Table). The two patients developed *C. auris* infections in 2017 (patient A) and 2018 (patient B), and multiple samples obtained from them had *C. auris*-positive cultures. Patient A had *C. auris* isolated from a central venous catheter tip and later from blood and urine cultures; patient B had *C. auris* isolated from a urine sample and a tracheal aspirate. All isolates were resistant to fluconazole; seven of 13 (54%) isolates from patient A and three of five (60%) isolates from patient B were resistant to amphotericin B; no isolates were initially resistant to echinocandins. Neither patient was known to have received antifungal medications before the diagnosis of *C. auris* infection, but both patients were treated with prolonged courses of echinocandins after *C. auris* was identified. Patient A was also treated with amphotericin B. Cultures taken after echinocandin therapy from both patients yielded *C. auris* isolates resistant to fluconazole, amphotericin B, and echinocandins. Both patients died; the role of *C. auris* in their deaths is unclear.

**TABLE:** Characteristics of three *Candida auris* cases with emergence of pan-resistance to antifungal agents — New York, 2019

Characteristic	Patient A	Patient B	Patient C
**Underlying condition**	Chronic ventilator dependence	Chronic ventilator dependence, alcohol dependence	Acute mechanical ventilation, alcohol dependence, chronic skin condition
**Antifungal medication received**	Echinocandin, amphotericin B	Echinocandin	Echinocandin
**Date pan-resistance confirmed**	February 2019	March 2019	June 2019*
**Sample type for pan-resistant isolate**	Blood	Urine	Rectal swab
**Time from first isolation of *C. auris* to collection of pan-resistant sample**	22 mos	13 mos	2 mos
**Time from isolation of pan-resistant *C. auris* to patient’s death**	2 wks	3–4 wks	10 mos
**MICs for pan-resistant isolates (*μ*g/mL)^†^**			
Triazole class			
Fluconazole	>256	>256	>256
Voriconazole	2	2	2
Posaconazole	0.25	0.5	0.25
Polyene class			
Amphotericin B	2	2	2
Echinocardin class			
Caspofungin	16	2	16
Anidulafungin	4	4	4
Micafungin	4	4	4
**No. of facilities at which screening was conducted**	1	2	1^§^
**No. of contacts with *C. auris*/No. tested (%)**	4/35 (11)	2/50 (4)	0/15^§^(0)
**No. of contacts with pan-resistant *C. auris***	0	0	0^§^
**No. of environmental surfaces and equipment with *C. auris*/No. tested (%)**	14/36 (39)	3/28 (11)	1/11^§^ (9)
**No. of environmental surfaces with pan-resistant *C. auris***	0	0	0^§^

**Abbreviation:** MIC = minimum inhibitory concentration.

* Isolate was from April 2017.

^†^ Tentative CDC MIC breakpoints (***µ*g/mL): fluconazole, ≥32; voriconazole: N/A; amphotericin B, ≥2; caspofungin, ≥2; anidulafungin ≥4; micafungin, ≥4.** https://www.cdc.gov/fungal/candida-auris/health-professionals.html***.***

^§^ Data from an assessment of contacts and environments in March 2017, approximately 1 month before collection of the pan-resistant isolate; laboratory surveillance of a sampling of *Candida* isolates from urine was also conducted.

No epidemiologic links were found between the two patients. They resided in and were patients at different health care facilities in the same borough of New York City, and neither patient had any known domestic or international travel. Point prevalence surveys, environmental sampling, and infection control assessments were performed at facilities where the two patients had resided to determine whether spread of the resistant isolates occurred (*2*,*4*,*5*). No pan-resistant isolates were identified among contacts or on environmental surfaces from the index patients’ rooms or common equipment (after discharge and terminal cleaning) at the three facilities that had cared for these two patients; however, non–pan-resistant *C. auris* was isolated from other patients and the environment at two of these facilities and from the environment at the third facility. Additional infection control and cleaning interventions were implemented by the facilities based on gaps identified during infection control assessments.

After identification of patients A and B in 2019, a retrospective review of all New York *C. auris* isolates and additional antifungal susceptibility testing at CDC identified a third patient (patient C), from whom a *C. auris* isolate from 2017 was found to be resistant to the three major antifungal classes. Patient C was also aged >50 years and had multiple comorbidities and a prolonged hospital admission and long-term care admission at facilities that were different (including in another borough) from those that cared for patients A and B. The initial isolate of *C. auris* from patient C was from a February 2017 blood culture; treatment with an echinocandin for 2 weeks followed. Serial isolates obtained from February to early April 2017 were resistant to fluconazole, had varying susceptibility to amphotericin B (11 of 17 [65%] total isolates resistant), and were initially susceptible to echinocandins; the isolate resistant to all three classes of antifungals was obtained from a rectal swab collected in late April 2017 to assess ongoing colonization following resolution of active infection. Patient C was discharged to a long-term care facility (different from the facilities that cared for patients A and B) on contact precautions. Subsequent serial surveillance cultures from several body sites were obtained, and all remained negative for >6 months until the patient died from underlying medical conditions. Patient C was not known to have had any recent foreign or domestic travel and did not have any known contact with patient A or patient B.

Isolates from all three patients were initially sensitive to echinocandins; resistance was detected after treatment, indicating that it emerged during treatment with the drugs. No evidence of transmission of the resistant isolates following these events was found.

## Discussion

The precise mechanism of resistance in these isolates is unknown, although echinocandin resistance in other species of *Candida* is linked to mutations in the drug target protein Fks1 (*7*). Approximately 3 years into the New York outbreak, these pan-resistant isolates still appear to be rare, but their emergence is concerning. In other countries with earlier emergence of *C. auris,* higher levels of echinocandin resistance and pan-resistance have been reported (*8*). An isolate from Illinois with development of echinocandin resistance after echinocandin treatment was recently described, although that isolate was susceptible to azoles (*9*). The pan-resistant cases reported here were all from New York, where the South Asia clade (clade 1) predominates (*5*). This clade is known to exhibit increased antifungal resistance compared to other clades of *C. auris* (*8*). Surveillance for additional pan-resistant isolates in New York is ongoing.

Echinocandins are the treatment of choice for *C. auris* infections (*1*). Most New York *C. auris* strains are fluconazole-resistant, and most strains of C. *auris* have been susceptible to echinocandins (*1*). However, because of the potential for development of resistance, patients on antifungal treatment for *C. auris* should be monitored closely for clinical improvement, and follow-up cultures should be obtained. Repeat susceptibility testing should also be conducted, especially in patients previously treated with echinocandins. Consultation with an infectious disease specialist is recommended, especially given the possibility of emergence of pan-resistance.

These findings illustrate the need to continue surveillance for *C. auris,* encourage prudence in the use of antifungal medications, and conduct susceptibility testing on all clinical isolates, including serial isolates from a single patient, especially those treated with echinocandins.

**Summary**

**What is already known about this topic?**

*Candida auris* is an emerging yeast that is often drug-resistant.

**What is added by this report?**

Three chronically ill patients in New York were identified as having pan-resistant *C. auris* after receipt of antifungal medications. No transmission of the pan-resistant isolates was found in patient contacts or the facility environments.

**What are the implications for public health practice?**

Three years after the first identification of *C. auris* in New York, pan-resistant isolates remain rare. Continued surveillance for *C. auris*, prudent antifungal use, and susceptibility testing for all *C. auris* clinical isolates (especially after patients have been treated with antifungal drugs) are needed.

## Footnotes

* E-test, previously known as Epsilometer test, is a method for antimicrobial susceptibility testing that provides an MIC.
